# Parameter advising for multiple sequence alignment

**DOI:** 10.1186/1471-2105-16-S2-A3

**Published:** 2015-01-28

**Authors:** Dan DeBlasio, John Kececioglu

**Affiliations:** 1 Department of Computer Science, University of Arizona, Tucson, AZ, 85721, USA

## Background

While the multiple sequence alignment output by an aligner strongly depends on the parameter values used for its alignment scoring function (i.e. choice of gap penalties and substitution scores), most users rely on the single default parameter setting. A different parameter setting, however, might yield a much higher-quality alignment for a specific set of input sequences. The problem of picking a good choice of parameter values for a given set of input sequences is called parameter advising. A *parameter advisor *has two ingredients: (i) a *set *of parameter choices to select from, and (ii) an *estimator *that estimates the accuracy of a computed alignment; the parameter advisor then picks the parameter choice from the set whose resulting alignment has highest estimated accuracy.

Our estimator Facet (**F**eature-based **Ac**curacy **E**s**t**imator) is a linear combination of real-valued feature functions of an alignment. We assume the feature functions are given as well as the universe of parameter choices from which the advisor's set is drawn. For this scenario we define the problem of learning an optimal advisor by finding the best possible parameter set for a collection of training data of reference alignments. Learning optimal advisor sets is NP-complete [1]. For the advisor sets problem, we develop a greedy $\frac{\ell }{k}$-approximation algorithm that finds near optimal sets of size at most *k *given an optimal solution of size ℓ<k. For the advisor estimator problem, we have an efficient method for finding the coefficients for the estimator that performs well in practice [2,3].

## Results

### Parameter advising

**Figure 1:**
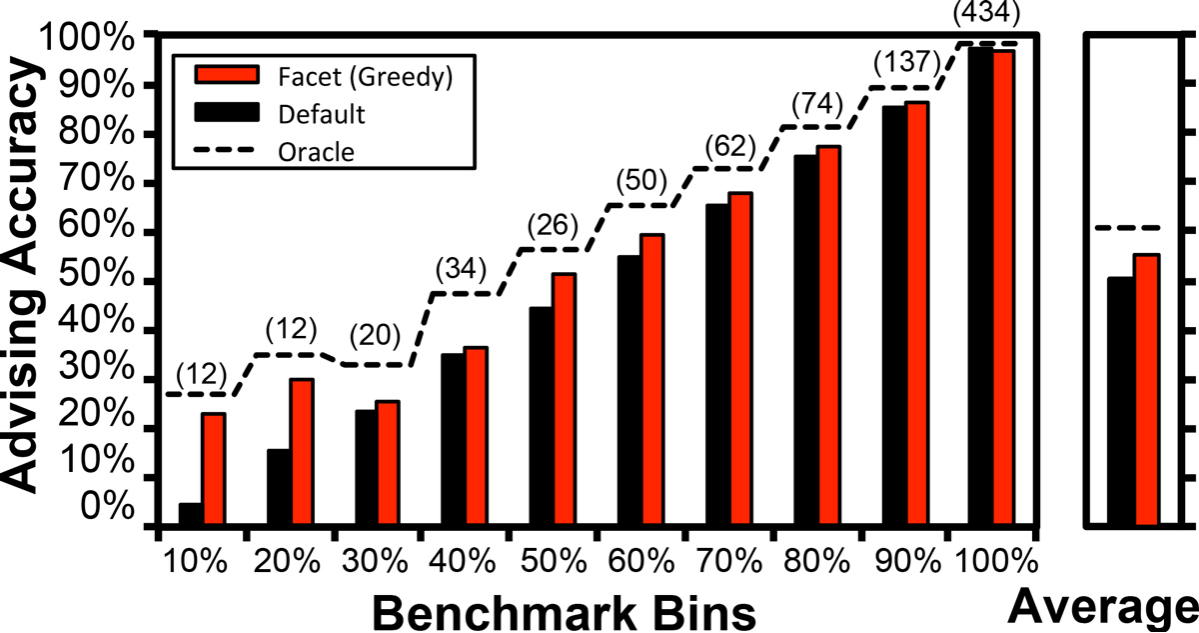
**Advising accuracy of Facet within benchmark bins**.

We apply parameter advising to boost the true accuracy of the Opal aligner [4,5], where the advisor is using parameter sets found by the $\frac{\ell }{k}$-approximation algorithm. Figure 1 shows the accuracy of the advisor for a parameter set of size *k = *10, where the benchmarks are assigned to bins based on their accuracy using a default parameter choice; the figure also shows the accuracies when using a single default parameter choice, and an oracle. The number of benchmarks per bin is indicated above the columns. An *oracle *is an advisor that knows the true accuracy of an alignment; its accuracy is shown by the dotted line, which gives the performance of a perfect advisor. Notice that in many cases the performance of the estimator is close to the oracle. This is most clear on the bin which has lowest average accuracy, where advising increases the average accuracy by almost 20% compared to using a single default parameter.

**Figure 2:**
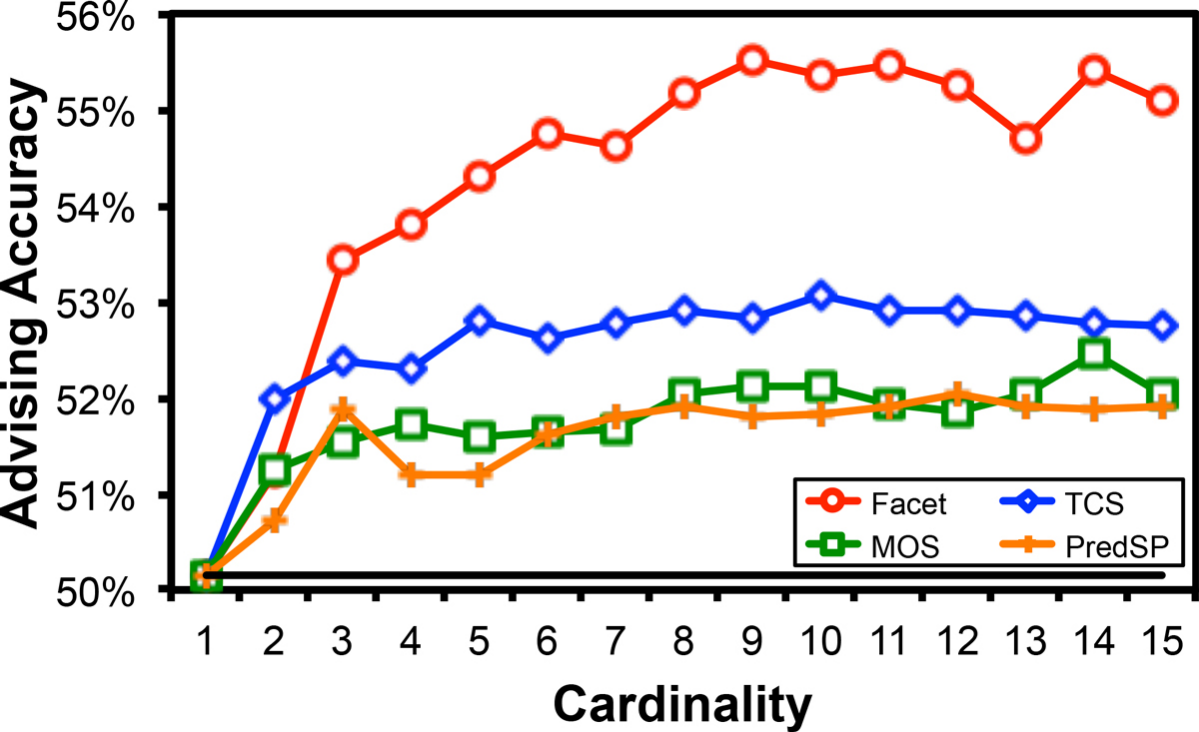
**Average advising accuracy of estimators on sets of varying cardinality**.

Figure 2 shows the average advising accuracy for parameter sets of various cardinalities using as the estimator Facet [3], TCS [6], MOS [7], and PredSP [8], where in the average, benchmark bins contribute equally. The vertical axis is advising accuracy on the testing data, averaged over all benchmarks and all folds using 12-fold cross-validation. The horizontal axis is the cardinality *k *of the greedy advisor set. Greedy advisor set found by the approximation algorithm are augmented from the exact set of cardinality ℓ = 1 (namely, the best single parameter choice). Notice that Facet (the topmost curve in the plot) continues to increase in advising accuracy up to cardinality *k *= 6. Notice also that while all of the advisors reach a plateau, for Facet this occurs at a greater cardinality and accuracy than for other estimators.

### Accuracy estimation

Our tool Facet (**F**eature-based **Ac**curacy **E**s**t**imator) [9] is an easy-to-use, open-source utility for estimating the accuracy of a protein multiple sequence alignment. Facet evaluates the estimated accuracy of a computed alignment as a linear combination of real-valued feature functions. We considered 12 features of which we found an optimal subset of 5 that provide the best performance for alignment advising. Many of the most useful features utilize information about protein secondary structure. We find coefficients by fitting the difference in estimator values to the difference in true accuracy for pairs of examples where the correct alignment is known. This "difference fitting" approach is computationally efficient and yields an estimator that works well for advising.

**Figure 3:**
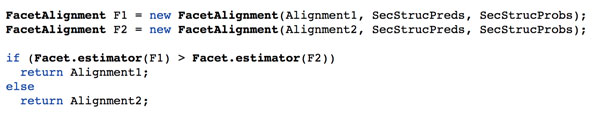
**Example of invoking Facet in Java**.

Facet is open-source software that allows users to estimate accuracy as either (1) a stand alone tool, or (2) a software library that can be integrated into a pre-existing Java application. The implementation provides optimized default coefficients and features. These coefficients may also be specified manually and new features can also be added. Figure 3 shows a simple example of using Facet within a Java application to choose between two alignments of the same set of sequences. The secondary structure predictions are computed on the unaligned sequences and can be reused between the two alignments.

The Facet website provides parameter sets that can be used with the Opal aligner (namely substitution matrices and affine gap penalties), as well as scripts for structure prediction.

## Conclusion

While the new problem of learning optimal parameter sets for an advisor is NP-complete, in practice our greedy approximation algorithm efficiently learns parameter sets that are remarkably close to optimal. Moreover, these parameter sets significantly boost the accuracy of an aligner compared to a single default parameter choice, when advising using the best accuracy estimators from the literature.
